# An ATL78-Like RING-H2 Finger Protein Confers Abiotic Stress Tolerance through Interacting with RAV2 and CSN5B in Tomato

**DOI:** 10.3389/fpls.2016.01305

**Published:** 2016-08-29

**Authors:** Jianwen Song, Yali Xing, Shoaib Munir, Chuying Yu, Lulu Song, Hanxia Li, Taotao Wang, Zhibiao Ye

**Affiliations:** ^1^Key Laboratory of Horticultural Plant Biology, Ministry of Education, Huazhong Agricultural UniversityWuhan, China; ^2^Key Laboratory of Horticultural Crop Biology and Genetic improvement (Central Region), Ministry of Agriculture, Huazhong Agricultural UniversityWuhan, China

**Keywords:** abiotic stress, ATL78L, COP9, CSN5B, RAV2, tomato

## Abstract

RING finger proteins play an important role in plant adaptation to abiotic stresses. In the present study, a wild tomato (*Solanum habrochaites*) cold-induced RING-H2 finger gene, *ShATL78L*, was isolated, which has been identified as an abiotic stress responsive gene in tomato. The results showed that *ShATL78L* was constitutively expressed in various tissues such as root, leaf, petiole, stem, flower, and fruit. Cold stress up-regulated *ShATL78L* in the cold-tolerant *S. habrochaites* compared to the susceptible cultivated tomato (*S. lycopersicum*). Furthermore, *ShATL78L* expression was also regulated under different stresses such as drought, salt, heat, wound, osmotic stress, and exogenous hormones. Functional characterization showed that cultivated tomato overexpressing *ShATL78L* had improved tolerance to cold, drought and oxidative stresses compared to the wild-type and the knockdown lines. To understand the underlying molecular mechanism of *ShATL78L* regulating abiotic stress responses, we performed yeast one-hybrid and two-hybrid assays and found that RAV2 could bind to the promoter of *ShATL78L* and activates/alters its transcription, and CSN5B could interact with *ShATL78L* to regulate abiotic stress responses. Taken together, these results show that ShATL78L plays an important role in regulating plant adaptation to abiotic stresses through bound by RAV2 and interacting with CSN5B.

**Highlight:** RAV2 binds to the promoter of *ShATL78L* to activates/alters its transcription to adapt the environmental conditions; furthermore, *ShATL78L* interacts with CSN5B to regulate the stress tolerance.

## Introduction

Plants are often exposed to a variety of abiotic stresses such as drought, temperature extreme, salinity, and hypoxia. These unfavorable environmental conditions negatively affect the plant growth, development, and productivity. Nevertheless, plants have evolved a series of intricate mechanisms and regulatory networks to respond and adapt to unfavorable environmental conditions. In recent years, more and more stress responsive genes have been identified using forward or reverse genetic approaches (Liu et al., 2012; Catala et al., 2014; Ding et al., 2015; Ma et al., 2015). It is noteworthy that transcription factors play an important role in stress tolerance (Chinnusamy et al., 2007). For instance, the R2R3-MYB transcription factor MYB15 negatively regulates the expression of CBFs and accelerates the freezing tolerance in *Arabidopsis* (Agarwal et al., 2006). The cotton WRKY transcription factor GhWRKY17 responds to drought and salt stresses through ABA signaling and regulates the cellular ROS production in plants (Yan et al., 2014). *Arabidopsis* transcription factor NAC016 triggers drought stress responses by repressing AREB1 transcription through a trifurcate feed-forward regulatory loop involving NAP (Sakuraba et al., 2015). In addition, RING finger proteins also play important role in plant stress responses. The RING finger ubiquitin E3 ligase SDIR1 targets SDIR1-INTERACTING PROTEIN1 to modulate the salt stress response and ABA signaling in *Arabidopsis* (Zhang et al., 2015). Another RING finger E3 ligase, STRF1, is a membrane trafficking-related ubiquitin ligase, which helps the plants to respond to salt stress by monitoring intracellular membrane trafficking and ROS production (Tian et al., 2015).

RING finger proteins are a special type of zinc finger proteins, which consist of 40–60 residues that binds two zinc atoms (Freemont, 1993). The RING finger domain appears to be a convenient scaffold that can be altered to provide functional specificity and the RING finger motif is defined as Cys-X2-Cys-X(9–39)-Cys-X(1–3)-His-X(2–3)-Cys/His-X2-Cys-X(4–48)-Cys-X2-Cys, where X is any amino acid (Borden and Freemont, 1996). Functional studies of the RING finger proteins revealed their wide range of roles in various biological processes including viral replication, signal transduction, and development (Tian et al., 2015). In contrast to two canonical RING types (C3H2C3 or C3HC4), additional types of modified RING domains including RING-V, RING-D, RING-S/T, RING-G, and RING-C2, were identified in *Arabidopsis* (Stone et al., 2005). In plants, different histidine/cysteine patterns correspond differently to C3H2C3 (RING-H2) and C3HC4 (RING-HC) RING-finger types. The consensus sequence of RING-H2 finger can be described as Cys-X2-Cys-X(9–39)-Cys-X(1–3)-His-X(2–3)-His-X2-Cys-X(4–48)-Cys-X2-Cys, whereas the C3HC4-type finger is defined as Cys-X2-Cys-X(9–39)-Cys-X(1–3)-His-X(2–3)-Cys-X2-Cys-X(4–48)-Cys-X2-Cys (Borden and Freemont, 1996).

In recent years, a large number of RING finger proteins have been identified in different plant species. For example, 688 RING domains in 663 predicted proteins and nine RING types were identified in apple (Li Y. et al., 2011). 187 ANK C3HC4-type RING finger proteins were identified from 29 plant species (Yuan et al., 2013). Meanwhile, more and more RING finger proteins have been reported to be involved in plant responses to biotic and abiotic stresses. A C3HC4 RING finger E3 ligase OsDIS1 negatively regulates the drought response through transcriptional regulation of different stress-related genes and possibly through posttranslational regulation of OsNek6 in rice (Ning et al., 2011). A C3HC4-type RING finger protein, EIRP1 E3 ligase positively regulates disease resistance in plants by mediating proteolysis of the negative regulator VpWRKY11 through degradation by 26S proteasome (Yu et al., 2013). The RING-type E3 ligase CaAIR1 plays a role in regulating ABA signaling and drought stress response in pepper (Park et al., 2015). Furthermore, RING-H2 finger proteins also play an important role in stress tolerance. Upregulation of an *Arabidopsis* RING-H2 gene, XERICO, confers drought tolerance through increased ABA biosynthesis (Ko et al., 2006). The RING-H2 finger E3 ubiquitin ligase, OsRFPH2-10, is involved in antiviral defense at early stages of rice dwarf virus infection (Liu et al., 2014).

In our previous microarray analysis of transcriptome differences between the cold tolerant (*Solanum habrochaites* LA1777 and its introgression line LA3969 under the genetic background of LA4024) and the sensitive (*S. lycopersicum* LA4024) genotypes under cold stress, a RING-H2 finger gene, *ATL78L*, was found to be more strongly induced in the two tolerant genotypes than in the sensitive one (Liu et al., 2012). However, little is known about the role of this gene in response to abiotic stresses in tomato. Moreover, the mechanism of drought and cold tolerance in tomato needs to be further investigated. In this study, we isolated the *S. habrochaites* RING-H2 finger gene, *ShATL78L*, and overexpressed this gene in *S. lycopersicum* cv. Ailsa Craig. The overexpression transgenic plants significantly improved drought and cold tolerance. Furthermore, we found that RAV2 can directly bind the promoter of *ShATL78L* and regulate its expression, and *ShATL78L* can interact with CSN5B to regulate abiotic stress responses in tomato. Our study provides new insights into the regulatory pathways involving *ShATL78L* in plant responses to various abiotic stresses.

## Materials and Methods

### Plant Materials and Growth Conditions

Seedlings of tomato wild relative (*S. habrochaites*) LA1777 were grown under a natural light in a greenhouse with temperature regimes of 24–28°C and relative humidity of approximately 70–80%. Roots, stems, leaves, flowers and fruits were collected from these plants for RNA isolation.

T_2_ generation of overexpression lines (OE7, OE8, OE9) and RNA interference (RNAi) line (Ri3), as well as the wild-type (*S. lycopersicum* cv. Ailsa Craig, AC), were tested for their tolerance to abiotic stresses. The abiotic stress treatments were imposed in the greenhouse. Seedlings were grown at 24–28°C and relative humidity of 70–80% of natural light. Four-week-old seedlings were used for stress tolerance assays.

### Abiotic Stress and Hormone Treatments

The cold tolerant *S. habrochaites* LA1777 was used for the analysis of *ATL78L* expression. Four-week-old seedlings were transferred to a growth chamber at 25 ± 2°C and relative humidity of 70% with a photoperiod of 14 h light/10 h dark and 200 μmol m^-2^s^-1^light intensity. After 3 days, seedlings were used for abiotic stress and hormonal treatments, viz., drought, cold, salt, heat, wound, polyethylene glycol6000 (PEG6000), mannitol, MV, ETH, SA and IAA. For drought stress, seedlings of LA1777 were washed with tap water thoroughly to obliterate substrates, and then dehydrated on filter papers. Cold stress treatment was performed by transferring seedlings of LA1777 to a growth chamber at 4°C under the same light condition as described above. For salinity treatment, seedlings of LA1777 were irrigated once with 200 mM NaCl solution (200 mL per pot). For heat treatment, seedlings were subjected to a high temperature of 40°C. For wound treatment, the leaves of the seedlings were pricked once with a needle. For hormone treatments, the seedling leaves were sprayed once with either 30% PEG6000, 200 mM mannitol, 0.2 mM ETH, 0.1 mM SA, or 0.1 mM IAA. One hundred milliliters of hormone solution was used for three plants. The third fully expanded leaves from the growing point were collected at indicated time points after treatments. Samples were collected for RNA isolation.

### Sequence Alignment and Phylogenetic Analysis

The multiple sequence alignments were conducted using MUSCLE^1^. The evolutionary history was inferred using the Neighbor-Joining method. The evolutionary distances were computed using the Poisson correction method with the units of the number of amino acid substitutions per site. All positions containing gaps and missing data were eliminated. Phylogenetic analyses were conducted in MEGA6 (Tamura et al., 2013). The sequences of the proteins were downloaded from EnsemblPlants^2^.

### Cloning and Analysis of *ShATL78L*

The full-length cDNA, gDNA and promoter sequences of *ATL78L* were obtained from The Sol Genomics Network^3^, and the gene identifier of *ATL78L* is Solyc11g005280. The primer sequences used in this study were listed in Supplementary Table S1. The homologous protein sequences of ShATL78-like were found from the NCBI^4^ and aligned using the DNAman software 5.2.2 (LynnonBiosoft). The PLACE database (Higo et al., 1999) was used to identify potential motifs in the *ShATL78L* promoter sequence.

### RNA Extraction and qRT-PCR

Total RNA was isolated using the TRIzol reagent (Invitrogen, USA). To remove genomic DNA contamination, the RNA was treated with DNase I at 37°C for 30 min. First strand cDNA was synthesized using the HiScript II 1st cDNA Synthesis Kit (Vazyme, China) according to the manufacturer’s protocol.

The expression of *ATL78L* under various treatments and in transgenic lines was analyzed using qRT-PCR, which was performed using SYBR^®^ Premix Ex Taq^TM^ (TaKaRa, China) and the LightCycler480 System (Roche, Switzerland) according to the supplier’s protocol. The PCR program was as follows: 95°C for 30 s; 40 cycles of 95°C for 5 s and 60°C for 25 s. Tomato actin was used as the internal control. All qPCR primers were designed by Primer5 and the primer sequences are listed in Supplementary Table S1. The qRT-PCR data of each gene were calculated using the 2^-ΔΔCT^ method (Livak and Schmittgen, 2001). qRT-PCR analysis was carried out with three technical replicates.

### Generation and Molecular Analysis of Transgenic Tomato Plants

The full-length open reading frame (ORF) of *ShATL78L* was amplified from the cDNA of LA1777 using the primers of *ATL78L* (Supplementary Table S1) designed according to the *S. lycopersicum* full-length cDNA. For *ATL78L* overexpression construct, the ORF of *ATL78L* was incorporated into the pDONR221 vector using the Clonase BP reaction (Invitrogen), and then the recombinant plasmid was incorporated into the pMV3 vector (modified from pHELLSGATE2) by the Clonase LR reaction (Invitrogen). For RNAi construct, a 135-bp fragment of *ATL78L* was amplified by *ATL78L*-Ri primers (Supplementary Table S1), and then incorporated into the pHGRV vector using the Clonase BP reaction (Invitrogen). All the expression vector constructs were transformed into *Agrobacterium* strain C58 by electroporation. Cotyledons of *S. lycopersicum* were used for transformation, as described previously (Ouyang et al., 2005).

Tomato T_0_ generation was checked by PCR using CaMV 35S promoter primer and *ATL78L* specific reverse primer (Supplementary Table S1). The expression of *ATL78L* in transgenic lines was analyzed by qRT-PCR. Three independent homozygous overexpression lines (OE7, OE8, and OE9) and one RNAi line Ri3 of *ShATL78L* were selected for further analyses.

### Abiotic Stress Tolerance Assays

For cold stress tolerance experiments, 4-week-old uniform-sized seedlings of T_2_ transgenic lines OE7, OE8, OE9, Ri3 and the wild-type (AC) were randomly chosen and grown in growth chamber with conditions of 4°C, 70% relative humidity and a 14 hr light/8 hr dark photoperiod (200 μmol m^-2^s^-1^ light intensity). The same conditions except at 25°C were used for control seedlings. After 7 days of cold treatments and 1 day of recovery, the third fully expanded leaves from the top were sampled.

For drought stress tolerance experiments, seedlings from the same stage of T_2_ transgenic lines (OE7, OE8, OE9, Ri3) and wild-type (AC) were placed in a greenhouse at 25°C under natural light conditions. Seedlings with drought treatment were withheld from water for 8 days, while the control seedlings were watered every 2 days. After 8 days of treatments, the third fully expanded leaves from the top of the plants were collected.

For salt and oxidative stress treatments, 4-week-old seedling of T_2_ transgenic lines OE7, OE8, OE9, Ri3 and wild-type AC were placed in a greenhouse at 25°C under natural light conditions, and the third fully expanded leaves from the top of the plants were collected. Fifteen leaf disks (0.8 cm) were detached from the third fully expanded leaves of each line and incubated in different concentrations of 15 ml solution with 0 mM NaCl, 100 mM NaCl and 5 μM MV for 72 h at 25°C, 70% relative humidity and a 16 h light/8 h dark photoperiod (200 μmol m^-2^s^-1^ light intensity) conditions before the Chl contents were measured.

### Measurements of Water Loss

For the water loss assay, the third fully expanded leaves from the top of the control and stressed plants were detached and used for relative dehydration rate determination. The leaves were placed on a filter paper with conditions of 25°C and 60% relative humidity and weighed after 0, 1, 2, 3, 6, 9, and 18 h. Ten compound leaves of each line were weighed. The relative drainage was calculated according to the formula: the relative drainage (%) = 100 × (W_0_ - W_t_)/W_0_, where *W*_0_ and *W*_t_ are weights after 0 h and t h, respectively.

### Protein Interaction Assay Using the Yeast Two-Hybrid System

The ORF of *ShATL78L* was amplified using specific primers ATL78L-BD (Supplementary Table S1) containing *Nde*I/*Pst*I restriction sites, thus corresponding to the yeast expression vector pGBKT7 (Clontech, USA), to construct pGBKT7-*ShATL78L*. The pGADT7-CSN5B was combined by the homologous recombination method (ClonExpress^TM^ II One Step Cloning Kit, Vazyme, China). According to the manufacturer’s protocol, the plasmids of pGBKT7-*ShATL78L*+pGADT7-*CSN5B*, pGBKT7-Lam+pGADT7-RecT (negative control), and pGBKT7-53+pGADT7-RecT (positive control) were co-transformed into yeast *Saccharomyces cerevisiae* strain AH109. The positive and negative controls were provided with the BD Matchmaker library construction and screening kits (Clontech, USA). The transformants were tested on the SD/-Leu/-Trp and the SD/-Ade/-His/-Leu/-Trp media. *CSN5B* accession numbers in the SGN database are Solyc06g073150 and Solyc11g017300.

### Binding Assay Using the Yeast One-Hybrid System

For the yeast one-hybrid assay, the promoter segments of *ShATL78L* were PCR amplified using the primers ATL78L-P (Supplementary Table S1) containing *Eco*RI/*Mlu*I restriction sites, so that the segments could be inserted into pHIS2 (Clontech, USA). Same as pGADT7-*CSN5B*, pGADT7-*RAV2* was also combined by homologous recombination. Plasmids pHIS2-ATL78LP+pGADT7-*RAV2*, p53HIS2+pGAD-Rec2-53 (positive control) and pHIS2-ATL78LP+pGADT7 (negative control) were co-transferred into yeast strain Y187, and the binding was detected on SD/-Leu/-Trp/-His selective plates containing 90 mM 3-AT (3-amino-1,2,4,-triazole) according to the manufacturer’s instruction. *SlRAV2* accession number in the SGN database is Solyc05g009790.

### Statistical Analyses

Statistical analysis was carried out using SigmaPlot 10, Excel, and SPSS. A significant difference between pairs of groups was analyzed by Student’s *t*-test.

## Results

### Sequence Analysis of *ShATL78L* Isolated from *S. habrochaites*

**FIGURE 1:**
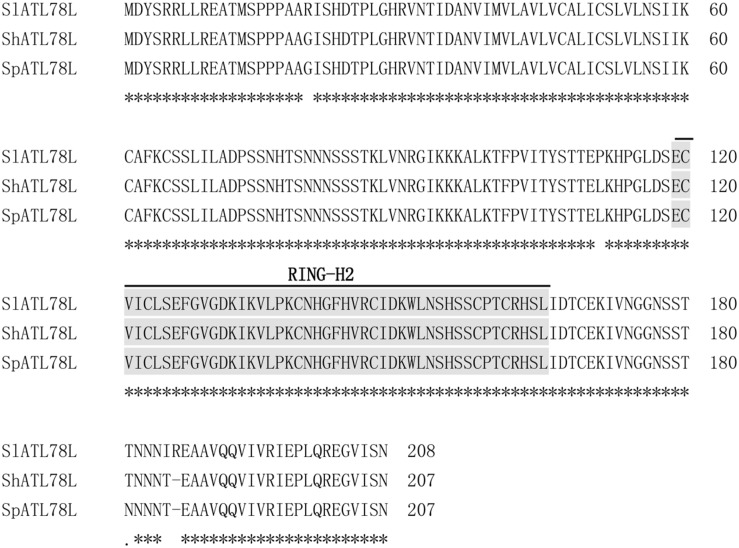
**Alignment of ATL78L amino acid sequences from *Solanum lycopersicum* cv. Ailsa Craig, *S. habrochaites* LA1777 and *S. pennellii* LA0716**. The RING-H2 finger domain is shown in gray background.

In our previous study, transcriptome profiles of cold tolerant (*S. habrochaites* LA1777 and its introgression line LA3969 under the genetic background of LA4024) and sensitive (*S. lycopersicum* LA4024) tomato genotypes were compared using microarray analysis, and one RING finger gene, *ATL78L*, was found to be more strongly induced by cold stress in the two tolerant genotypes than in the sensitive one (Liu et al., 2012). The complete ORFs of *ATL78L* were isolated from tomato Ailsa Craig (*S. lycopersicum*) and wild species LA1777 (*S. habrochaites*), as well as the wild species LA0716 (*S. pennellii*) that displays extremely high stress tolerance (Rick and Tanksley, 1981). The ORFs of *ShATL78L* and *SpATL78L* had the same size, consisting of 624 nucleotides and encoding 207 amino acid residues. Multiple alignments of amino acid sequences revealed that these three sequences showed a high degree of similarity to each other. Three amino acids at positions 20, 111, and 185 were different and one amino acid indel was found among the three genotypes (**Figure 1**). These four amino acids may functionally contribute to tomato stress tolerance.

ATL78L contains a RING finger domain (Cys-X2-Cys-X(9–39)-Cys-X (1–3)-His-X (2–3)-His-X2-Cys-X(4–48)-Cys-X2-Cys) and is classified as a RING-H2 finger protein (**Figure 1**). Phylogenetic analysis indicated that *SlATL78L* had nine paralogs in the cultivated tomato (*S. lycopersicum*) and showed the highest homology with PGSC0003DMT400015477 from potato (*S. tuberosum*). The closest homologue of *SlATL78L* from *Arabidopsis* is AT1G49230.1 (*ATL78*) therefore, Solyc11g005280 was named as *ATL78L* (Supplementary Figure S1).

### Differential Expression Pattern of *ATL78L* in Different Species of Tomato

**FIGURE 2:**
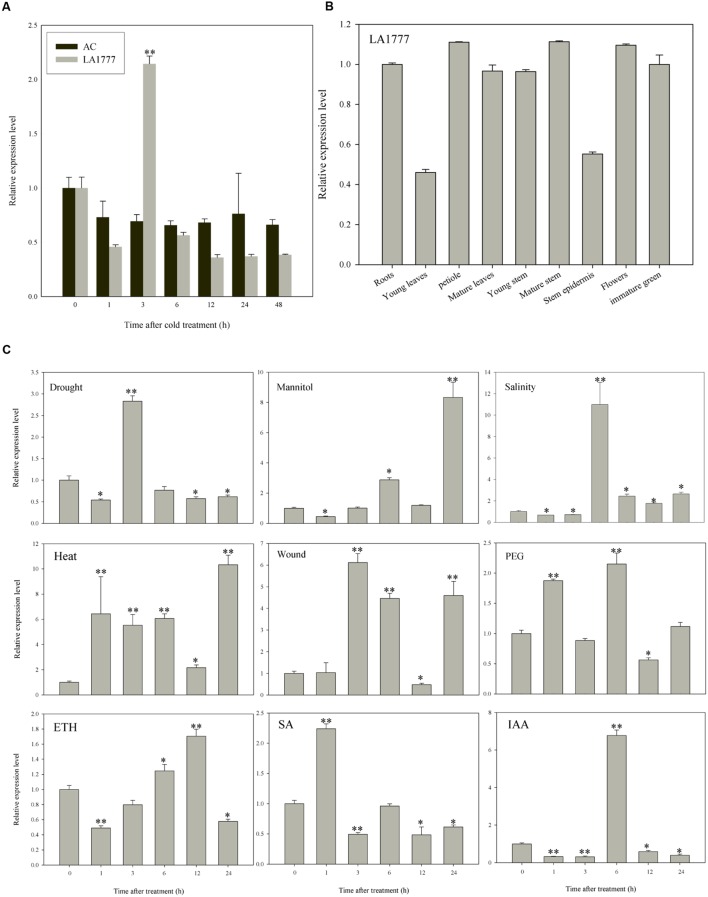
**Expression patterns of tomato *ATL78L* in different tissues or under various abiotic stresses and hormonal treatments**. **(A)** Expression levels of *ATL78L* in *Solanum habrochaites* LA1777 and *Solanum lycopersicum* cv. Ailsa Craig under cold stress. Asterisks indicate significant differences between LA1777 and AC (^∗^*P* < 0.05; ^∗∗^*P* < 0.01, Student’s *t*-test). **(B)** Expression pattern of *ShATL78L* in different tissues of LA1777 plants. **(C)** Expression patterns of *ShATL78L* in LA1777 seedlings under drought, salt, heat, wound, mannitol, PEG, ETH, SA, and IAA treatments. Asterisks indicate a significant difference (^∗^*P* < 0.05; ^∗∗^*P* < 0.01, Student’s *t*-test) compared with the corresponding controls. Four-week-old seedlings were treated by 4°C, 200 mM NaCl, 40°C, 200 mM mannitol, 30% PEG6000, 0.2 mM ETH, 0.1 mM SA or 0.1 mM IAA, respectively, for indicated time points. Values are means ± SE of three biological replicates. Each replicated sample consists of leaves from three seedlings.

To confirm the microarray results, expression levels of *ATL78L* in LA1777 and AC were examined under cold stress treatment. cDNAs of *ShATL78L* and *SlATL78L* were isolated to perform the expression analysis. As shown in **Figure 2A**, *ShATL78L* transcripts exhibited a significant increase after 3 h of cold stress treatment. However, no significant difference was observed in the relative expression level of *SlATL78L* between cold-treated and control plants. Therefore, there is a positive correlation between *ShATL78L* transcripts and cold tolerance in tomato. To further understand the role of the *ShATL78L* in different abiotic stresses, we investigated the expression pattern of *ShATL78L* in LA1777 under different abiotic stresses and hormonal treatments. As shown in **Figure 2C**, the transcript levels of *ShATL78L* were increased in response to drought, salinity, heat, wound, mannitol, PEG, ETH, SA, and IAA treatments. *ShATL78L* was strongly induced by drought, mannitol, salinity, heat, wound, and IAA treatments. A statistically similar but lower induction level was detected in response to SA, ETH, and PEG. To investigate whether the expression of *ShATL78L* has any tissue/organ specificity, the expression level of *ShATL78L* in different tissues of LA1777 was determined by qRT-PCR. As shown in **Figure 2B**, *ShATL78L* was constitutively expressed in all examined tissues with a little lower expression levels in young leaves and stem epidermis compared with other tissues.

### Molecular Characterization of *ShATL78L* Transgenic Plants

**FIGURE 3:**
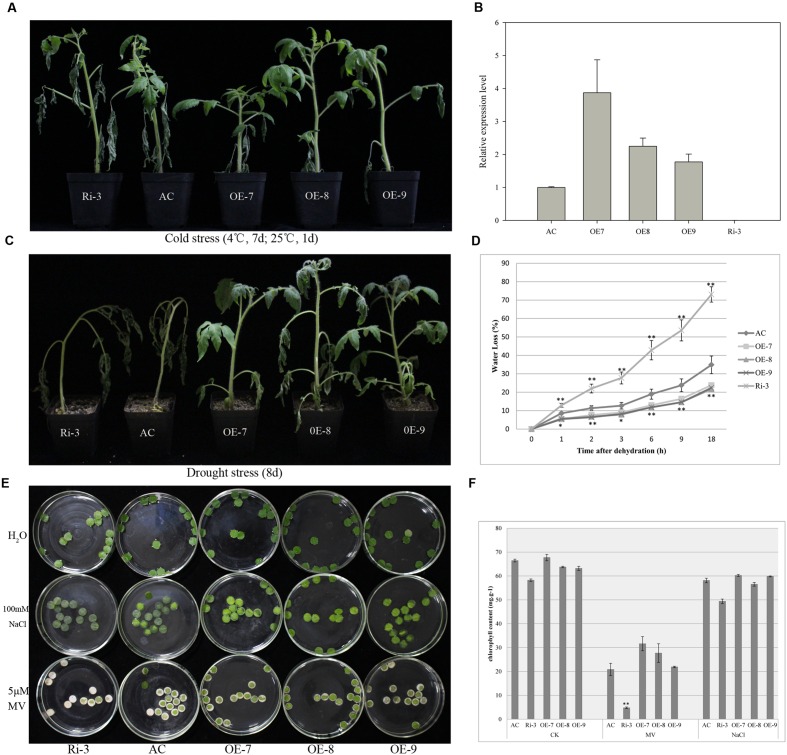
**Overexpression of *ShATL78L* enhanced the tolerance to multiple abiotic stresses in tomato**. **(A)** Phenotypes of seedlings from transgenic and wild-type tomato plants under cold stress conditions. Four-week-old seedlings of transgenic lines (T2 generation) and wild-type were treated at 4°C for 7 days and then recovered at 25°C for 1 day. **(B)** Relative expression level of *ATL78L* in the wild-type and transgenic lines. **(C)** Phenotypes of seedlings from transgenic lines and wild-type under drought stress conditions. Four-week-old seedlings of transgenic lines (T_2_ generation) and wild-type were withheld from water for 8 days. **(D)** Water loss of the detached leaves from wild-type and transgenic plants. **(E)** Leaf disks obtained from 4-week-old wild-type and transgenic seedlings were incubated in different solution of H_2_O, 100 mM NaCl and 5 μM MV for 72 h. **(F)** Relative chlorophyll content after the 100 mM NaCl and 5 μM MV treatment of the leaf disks. Data in **(B,D,F)** are means ± SE of three biologically replicated samples. AC represents the wild-type. OE-7, OE-8, OE-9, and Ri-3 represent three independent *ShATL78L* overexpression lines and the RNAi line, respectively. Asterisks indicate significant differences between transgenic lines and wild-type (^∗^*P* < 0.05; ^∗∗^*P* < 0.01, Student’s *t*-test).

In order to characterize the function of *ShATL78L*, the full-length ORF of *ShATL78L* was cloned into the plant expression vector pMV3 under the control of CaMV35S promoter. This construct was introduced into AC (*S. lycopersicum*). A total of 28 independent transformants (T_0_) were generated, and 23 of them were positive as confirmed by PCR analysis using CaMV35S forward and gene-specific reverse primer sets. At the same time, the RNAi lines were also generated through knocking down the ATL78L transcripts. Transgene expression level was examined by qRT-PCR. As shown in **Figure 3B**, four independent lines, OE-7, OE-8, OE-9, and Ri-3 displayed expected expression levels of *ATL78L*.

### Overexpression of *ShATL78L* Enhances Tomato Plant Tolerance to Cold and Drought Stresses

To characterize the functional role of *ShATL78L* in cold stress tolerance, 4-week-old seedlings of the four transgenic lines (T_2_) and the wild-type AC were examined under cold stress treatment at 4°C for 7 days, and then allowed to recover at 25°C for 1 day. After the stress treatment, seedlings of the AC line exhibited severe wilting, whereas the overexpression lines (OE-7, OE-8, OE-9) displayed much less leaf wilting symptoms, and the RNAi line Ri-3 showed higher leaf wilting symptoms compared to AC (**Figure 3A**). These findings demonstrated that *ShATL78L* plays a positive role in cold stress tolerance.

To investigate whether *ShATL78L* has an effect on drought stress tolerance, 4-week-old seedlings of the three overexpression lines (OE7, OE8, and OE9), the RNAi line (Ri-3) and the wild-type AC were withheld from water for eight consecutive days. After the drought stress treatment, obvious dehydration symptoms (leaf wilting) were found both in the RNAi line and wild-type plants compared to overexpression lines, which exhibited inconspicuous wilting. The stem of wild-type and RNAi seedlings bent due to loss of turgor pressure while the overexpression plants remained turgid and erect (**Figure 3C**). Water loss assay was conducted under the dehydration stress. The detached leaf water loss was significantly lower in the overexpression lines compared to wild-type plants while RNAi line showed the maximum water loss among all the treated lines (**Figure 3D**). These findings demonstrated that *ShATL78L* plays a positive role in drought stress tolerance.

### Overexpression of *ShATL78L* Improves Tomato Oxidative Stress Tolerance

The potential role of *ShATL78L* under salt and oxidative stress was further evaluated by incubating the leaf disks of 4-week-old seedlings in 100 mM NaCl and 5 μM MV solutions, respectively. After 72 h treatment, the leaf disks of the transgenic plants were similar to that of the wild-type AC in H_2_O and NaCl solutions. In the MV solutions, much more severe bleaching was observed in the RNAi line than overexpression plants, and wild-type plants showed an intermediate phenotype (**Figure 3E**). The Chl contents further confirmed the difference of salt and oxidative damages between transgenic and wild-type AC plants (**Figure 3F**). Taken together, these results supported that *ShATL78L* plays a positive role in tolerance to oxidative stress.

### Transcription Factor SlRAV2 (ABI3/VP1-Like) Specifically Binds to the Promoter of *ShATL78L*

**FIGURE 4:**
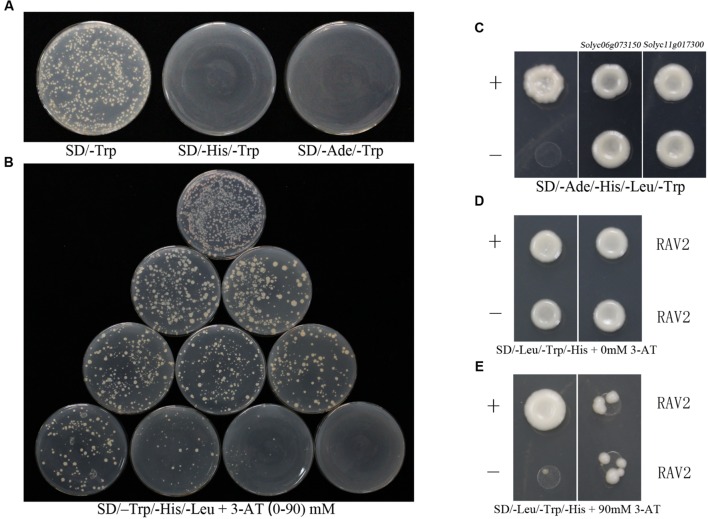
**CSN5B interacts with ATL78L whose promoter is bound by RAV2**. **(A)** Self-activation assay of *ATL78L.* The yeast cells containing pGBKT7-*ShATL78L* transformants were tested on SD/-Trp, SD/-His/-Trp, and SD/-Ade/-Trp media. **(B)** Screen the concentration of 3-AT for the yeast one-hybrid system. The yeast cells containing pHIS2-ATL78LP were examined on the SD/–Trp/-His/-Leu media with the 0 to 90 mM 3-AT, respectively. **(C)** ATL78L interacts with two CSN5B proteins. The yeast cell transformants containing pGBKT7-53+pGADT7-RecT (positive control), pGBKT7-Lam+pGADT7-RecT (negative control) and two pGBKT7-*ShATL78L*+pGADT7-*CSN5B* were tested on the SD/-Ade/-His/-Leu/-Trp media. **(D,E)** RAV2 binds to the promoter of *ATL78L*. Plasmids p53HIS2+pGAD-Rec2-53 (positive control), pHIS2-*ATL78LP*+pGADT7 (negative control) and pHIS2-ATL78LP+pGADT7-*SlRAV2* (the right column) were co-transferred into yeast strain Y187, the interactions were detected on SD/-Leu/-Trp/-His with 0 mM 3-AT **(D)** and 90 mM 3-AT **(E)**.

The induction of *ShATL78L* gene under various abiotic stresses prompted us to analyze the promoter of this gene (approximately 1000 bp upstream the transcription start site). The promoter sequence was isolated from LA1777 and fused with pHIS2 to construct the bait vector (pHIS2-ATL78LP). As shown in **Figure 4B**, 90 mM inhibitory 3-amino-1,2,4-triazole (3-AT; Sigma) was screened from 0 to 90 mM concentrations of 3-AT for the yeast one-hybrid system. Bait constructs and tomato cDNA library fusion plasmid were co-transformed into yeast strain Y187 and a positive clone of *SlRAV2* was selected from 152 clones for further confirmation (Supplementary Table S2). As shown in **Figure 4D**, all yeast cells harboring different constructs could grow on SD/-Trp/-His/-Leu without 3-AT, which indicated that all the recombinant plasmids were introduced successfully into yeast. However, co-transformed cells with pGADT7-SlRAV2 and pHIS2-ATL78LP could grow in the presence of the 90 mM 3-AT. Moreover, the growth of transformants containing constructs but lacking SlRAV2 was completely inhibited (**Figure 4E**), which suggested that SlRAV2 can bind to the promoter of *ATL78L* in yeast.

### *ShATL78L* Interacts with CSN5B *In vivo*

To further investigate the molecular mechanism of the *ATL78L*-mediated pathway in tomato stress tolerance, a yeast two-hybrid screening was performed to identify the interacting proteins with ATL78L. As shown in **Figure 4A**, *ATL78L* does not contain self-activation due to that the yeast cells pGBKT7-*ShATL78L* could not grow in the SD/-His/-Trp or SD/-Ade/-Trp media while could grow in the SD/-Trp medium. In the Y2H assay, the full-length ORF of *ShATL78L* was used as the bait to screen a tomato cDNA Y2H library, which was constructed with the prey vector pGADT7-Rec. Two positive clones of COP9 signalosome (CSN) complex subunit *CSN5B* were selected from 138 clones for further analysis (Supplementary Table S3). The amino acid sequences of these two genes showed high homology (Supplementary Figure S2). As shown in **Figure 4C**, the yeast cells co-transformed with pGBKT7-*ShATL78L* and pGADT7-*CSN5B* could grow in the SD/-Ade/-His/-Leu/-Trp media, same as the positive control (pGBKT7-53+pGADT7-RecT). In addition, negative control transformants (pGBKT7-Lam+pGADT7-RecT) did not grow in the same media. These results indicated that *ShATL78L* interacts with SlCSN5B in yeast.

## Discussion

### RING-H2 Finger Gene *ShATL78L* is Regulated by ETH, SA, and IAA

RING finger family proteins have been widely reported to play an important role in abiotic and biotic stresses (Lee et al., 2001; Cheung et al., 2007; Hong et al., 2007; Qin et al., 2008; Zhang et al., 2015). According to the motifs, *ATL78L* can be classified into RING-H2, a subgroup of RING finger proteins (Borden and Freemont, 1996). RING-H2 finger proteins are involved in different abiotic stress signaling pathways in plants (Ko et al., 2006; Zhang et al., 2007). However, the functions of RING-H2 proteins in tomato remain largely unknown (Hondo et al., 2007; Qi et al., 2016). Particularly, the molecular mechanism of RING-H2 finger proteins in response to stress tolerance is still unclear. *ShATL78L* has not been functionally characterized, though it was screened in our previous microarray experiment under cold stress (Liu et al., 2012). In this study, we showed that *ShATL78L* exhibited a strong response to cold, drought, salt, heat and wound stresses (**Figure 2C**).

It has been well reported that RING finger proteins can modulate various plant hormone responses, such as ABA (Ko et al., 2006; Zhang et al., 2007; Li H. et al., 2011), IAA (Gray et al., 2002; Xie et al., 2002), GA (Park et al., 2013), ETH (He et al., 2005), JA (Hondo et al., 2007), and SA (Cheong et al., 2009). Phytohormones play critical roles in plant adaptation to adverse environmental conditions. Cross-talk between different plant hormones and the environment results in synergistic or antagonist interactions that play a significant role in plant responses to abiotic stresses (Peleg and Blumwald, 2011). Our results indicated that *ShATL78L* was induced by different plant hormone treatments such as ETH, IAA, and SA (**Figure 2C**), which indicates that *ShATL78L* possibly functions as a balance regulator through mediating different signaling pathways between ETH, IAA, and SA in response to abiotic stresses.

### *ShATL78L* Enhances Multiple Abiotic Stress Tolerance in Tomato

In this study, we demonstrated that *ShATL78L* overexpression plants positively contributed to enhanced cold, drought, and oxidative stress tolerance (**Figures 3A,C,E**). These results are consistent with previous studies in other plant species (Hirayama and Shinozaki, 2010). On the other hand, suppression of the RING E3 ubiquitin ligase *AtATL78* increased the cold stress tolerance, while decreased drought stress tolerance (Kim and Kim, 2013). *Arabidopsis* RING-H2 gene *XERICO* confers drought stress tolerance through increased ABA biosynthesis (Ko et al., 2006). In *S. pimpinellifolium*, the RING Finger E3 Ligase *SpRing* is a positive regulator of salt stress signaling (Qi et al., 2016). However, *ShATL78L* transgenic plants did not show significantly different response to salt stress compared to the wild type (**Figure 3E**).

### *ShATL78L* is Directly Regulated by *RAV2*

**FIGURE 5:**
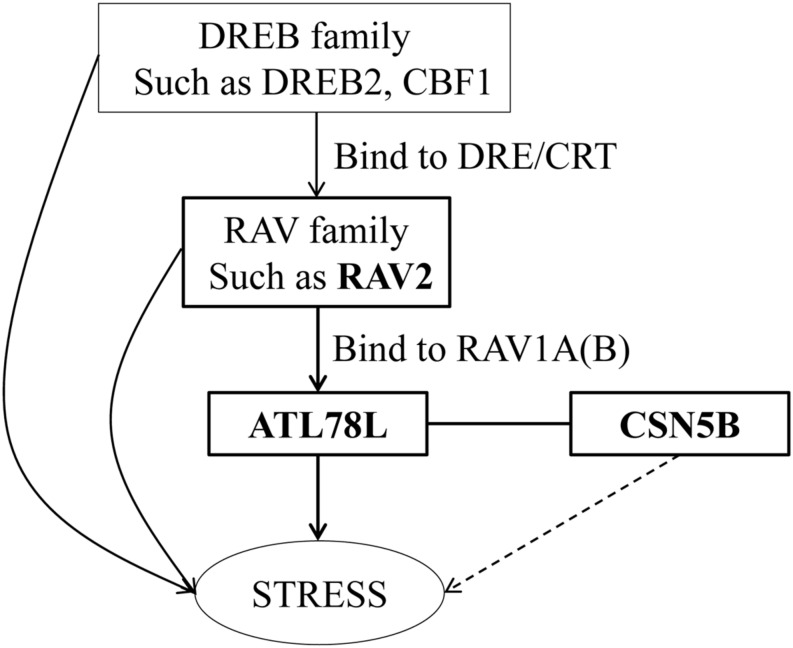
**Proposed role of *ShATL78L* in the plant stress signaling pathway**. The model shows the genetic interactions between *RAV2, ATL78L*, and *CSN5B* genes in regulating the responses to abiotic stress in tomato.

In this study, we demonstrate that RAV2 binds to the promoter of *ShATL78L* and activates/alters *ShATL78L* transcription (**Figures 4D,E**). The RAV transcription factor contains AP2-like and B3-like domains, which specifically bind to DNA with bipartite sequence motifs of RAV1-A (CAACA) and RAV1-B (CACCTG; Kagaya et al., 1999). The motif of RAV1-A and RAV1-B can be detected in the promoter of *ShATL78L*. It has been well reported that RAV2 responds to different abiotic stresses (Li C.W. et al., 2011; Fu et al., 2014; Matias-Hernandez et al., 2014; Mittal et al., 2014). The promoter of *RAV2*, which contains the CRT/DRE element, can be bound by the DREB transcription factors (Yamaguchi-Shinozaki and Shinozaki, 1994; Dubouzet et al., 2003; Li C.W. et al., 2011). DREB proteins specifically bind to DRE/CRT element and activate target gene transcription in *Arabidopsis* (Liu et al., 1998) and tomato (Li C.W. et al., 2011; Li et al., 2012). It has been well studied that DREB family play an important role in abiotic stresses in *Arabidopsis*, such as DREB (Liu et al., 1998) and CBF1 (Stockinger et al., 1997). In recent years, several studies have supported that DREB family genes exhibit similar role in abiotic stresses in tomato. For example, *SlDREB* gene acts as a positive regulator of drought stress responses by restricting leaf expansion and internode elongation (Li et al., 2012); The dehydration-responsive transcription factor *SlDREB2* mediates salt stress tolerance in tomato and *Arabidopsis* (Hichri et al., 2016). Tomato has a complete CBF cold responsive pathway, however, its CBF regulon differs from *Arabidopsis* and shows much smaller and less diverse in function (Zhang et al., 2004). Our results and these previous findings clearly indicate the responsiveness of *ShATL78L* to multiple abiotic stresses (**Figure 5**).

### *ShATL78L* Interacts with COP9 Signalosome Complex Subunit CSN5B in Response to Abiotic Stresses

COP9 (constitutive photomorphogenesis) is a component of the signaling complex mediating light control of development in *Arabidopsis* (Wei et al., 1994). CSN consists of eight subunits (CSN1-8), and plays a critical role in the ubiquitin/26S proteasome proteolytic regulatory pathway (Rubio et al., 2005). We show that *ShATL78L* interacts with CSN5B (**Figure 4C**), which plays a crucial role in abiotic stress tolerance (Wang et al., 2013). In rat, CSN5 inhibits cardiac L-type Ca^2+^ channel activity through protein–protein interactions (Kameda et al., 2006). Taken together, we hypothesize that *ShATL78L* responds to multiple abiotic stresses possibly through interacting with CSN5B, which alters the Ca^2+^ channel activity. On the other hand, CSN regulates plant growth and development through mediating protein degradation (Serino and Deng, 2003). In addition, CSN plays a vital role in mediating E3 ubiquitin ligase-mediated responses (Schwechheimer et al., 2001; Cavadini et al., 2016). Altogether, our results indicate that *ShATL78L* enhances multiple abiotic stresses tolerance possibly through altering the ubiquitin-mediated protein degradation (**Figure 5**).

## Conclusion

In conclusion, this study provides a possible molecular mechanism of *ShATL78L* in enhancing multiple abiotic stress tolerance in tomato (**Figure 5**). Our results clearly indicate that RAV2 binds to the promoter of *ShATL78L* and activates/alters transcription levels of *ShATL78L* in helping plants to adapt unfavorable environmental conditions. Meanwhile, *ShATL78L* interacts with CSN5B to regulate stress tolerances. However, further studies are still required to explore the detailed mechanism of COP9-mediated stress signaling in tomato.

## Author Contributions

JS designed and performed experiments, analyzed the data and drafted the manuscript. YX designed and conducted the qRTPCR experiments, and performed expression analysis. SM, CY, and LS designed and carried out the stress experiments. ZY, TW, and HL designed all the experiments and revised the manuscript.

## Conflict of Interest Statement

The authors declare that the research was conducted in the absence of any commercial or financial relationships that could be construed as a potential conflict of interest.

## Footnotes

1 http://www.ebi.ac.uk/Tools/msa/muscle/

2 http://plants.ensembl.org/index.html

3 http://solgenomics.net/

4 http://blast.ncbi.nlm.nih.gov/Blast.cgi

## ABBREVIATIONS

3-AT: 3-amino-1,2,4-triazole
ABA: abscisic acid
Chl: chlorophyll
DREB: dehydration-responsive element binding
ETH: ethephon
IAA: indole-3-acetic acid
MV: methyl viologen
PEG: polyethylene glycol
qRT-PCR: quantitative real-time PCR
SA: salicylic acid
