# Supplementary material for: An ATL78-Like RING-H2 Finger Protein Confers Abiotic Stress Tolerance through Interacting with RAV2 and CSN5B in Tomato
Source: Front Plant Sci. 2016 Aug 29;7:1305. doi: 10.3389/fpls.2016.01305 (PMC5002894; doi:10.3389/fpls.2016.01305)
Supplement: Supplementary file 1 [file Table_1.PDF]

Table S1. The primers used in this study

Abbreviation	Primer sequence (5′-3′)	Description
ATL78L-OE-FW	AAAAAGCAGGCTATGGACTATTCAAGGAGATTACTCCG	Vector construction primer
ATL78L-OE-RV	AGAAAGCTGGGTTTAATTGGATATCACACCTTCACGTT	
ATL78L-Ri-FW	AAAAAGCAGGCTCAGAATCAGCCACGATACACCAC	
ATL78L-Ri-RV	AGAAAGCTGGGTTTAAATGCACATTTTATGATGGAGTTC	
attB1	GGGGACAAGTTTGTACAAAAAAGCAGGCT	
attB2	GGGGACCACTTTGTACAAGAAAGCTGGGT	
35S	ACGCACAATCCCACTATCCTTC	CaMV 35S promoter specific primer
ATL78L-FW	ATGGACTATTCAAGGAGATTACTCCG	The cDNA and gDNA primer
ATL78L-RV	TTAATTGGATATCACACCTTCACGTT	
Qactin-Fw	GTCCTCTTCCAGCCATCCA	Q-PCR standard control primer (Accession No. BT013524)
Qactin-Rv	ACCACTGAGCACAATGTTACCG	
ATL78L-Q-FW	CCCTCATTGACACTTGCGAA	Q-PCR primer
ATL78L-Q-RV	TACTATAACTTGTTGTACTGCTGCCTC	
ATL78L-BD-FW	CATATGATGGACTATTCAAGGAGATTACTCCG	Yeast two-hybrid primer
ATL78L-BD-RV	CTGCAGCTTAATTGGATATCACACCTTCACGTT	
CSN5B1-AD-FW	GCCATGGAGGCCAGTGAATTCATGGACTCTCTGAATTCTTACGCA	
CSN5B1-AD-RV	CAGCTCGAGCTCGATGGATCCTCAGCTTTCGATCATGGGCT	
CSN5B2-AD-FW	GCCATGGAGGCCAGTGAATTCATGGACGCTCTGAATTCTTACGC	
CSN5B2-AD-RV	CAGCTCGAGCTCGATGGATCCTCAGGTTTCGACCATCGGC	
ATL78L-P2-FW	GAATTCTGCCCCTTTACATTAATCAAGAGA	Yeast one-hybrid primer
ATL78L-P2-RV	ACGCGTGGCTTCCCGGAGTAATCTCC	
RAV1-AD-FW	GCCATGGAGGCCAGTGAATTCATGGAAGGAAGTATTAGTAGCATAGATCA	
RAV1-AD-RV	CAGCTCGAGCTCGATGGATCCTTACAAAGCATCAATAATAACCCTTTG	
