# Supplementary material for: An ATL78-Like RING-H2 Finger Protein Confers Abiotic Stress Tolerance through Interacting with RAV2 and CSN5B in Tomato
Source: Front Plant Sci. 2016 Aug 29;7:1305. doi: 10.3389/fpls.2016.01305 (PMC5002894; doi:10.3389/fpls.2016.01305)
Supplement: Supplementary file 2 [file Table_2.DOCX]

| Table S2.List of genes identified by yeast one-hybrid. | |
| --- | --- |
|  |  |
| Gene ID | Annotation |
| Solyc01g005470 | Cell number regulator 10 |
| Solyc01g086870 | BHLH transcription factor |
| Solyc01g086940 | Zinc finger CCCH domain-containing protein 44 |
| Solyc03g118840 | RING-finger protein like |
| Solyc04g071610 | Water-stress inducible protein 3 |
| Solyc05g009790 | Transcription factor |
| Solyc09g007490 | Cell number regulator 8 |
| Solyc09g075420 | Ethylene responsive transcription factor 2b |
| Solyc12g056860 | BZIP transcription factor |
