# Supplementary material for: An ATL78-Like RING-H2 Finger Protein Confers Abiotic Stress Tolerance through Interacting with RAV2 and CSN5B in Tomato
Source: Front Plant Sci. 2016 Aug 29;7:1305. doi: 10.3389/fpls.2016.01305 (PMC5002894; doi:10.3389/fpls.2016.01305)
Supplement: Supplementary file 3 [file Table_3.DOCX]

| Table S3.List of genes identified by yeast double-hybrid. | |
| --- | --- |
|  |  |
| Gene ID | Annotation |
| Solyc00g005050 | Arabinogalactan protein |
| Solyc02g067760 | MYB transcription factor |
| Solyc04g007900 | Cell number regulator 2 |
| Solyc04g008560 | Unknown Protein |
| Solyc04g009950 | Pre-mRNA splicing factor |
| Solyc04g078760 | Protein binding protein |
| Solyc06g073150 | COP9 signalosome complex subunit 5b |
| Solyc07g007790 | Sucrose phosphate synthase |
| Solyc07g045290 | Long-chain-fatty-acid--CoA ligase 3 |
| Solyc11g017300 | COP9 signalosome complex subunit 5b |
