# Supplementary material for: An ATL78-Like RING-H2 Finger Protein Confers Abiotic Stress Tolerance through Interacting with RAV2 and CSN5B in Tomato
Source: Front Plant Sci. 2016 Aug 29;7:1305. doi: 10.3389/fpls.2016.01305 (PMC5002894; doi:10.3389/fpls.2016.01305)
Supplement: Supplementary file 4 [file Image_1.PDF]

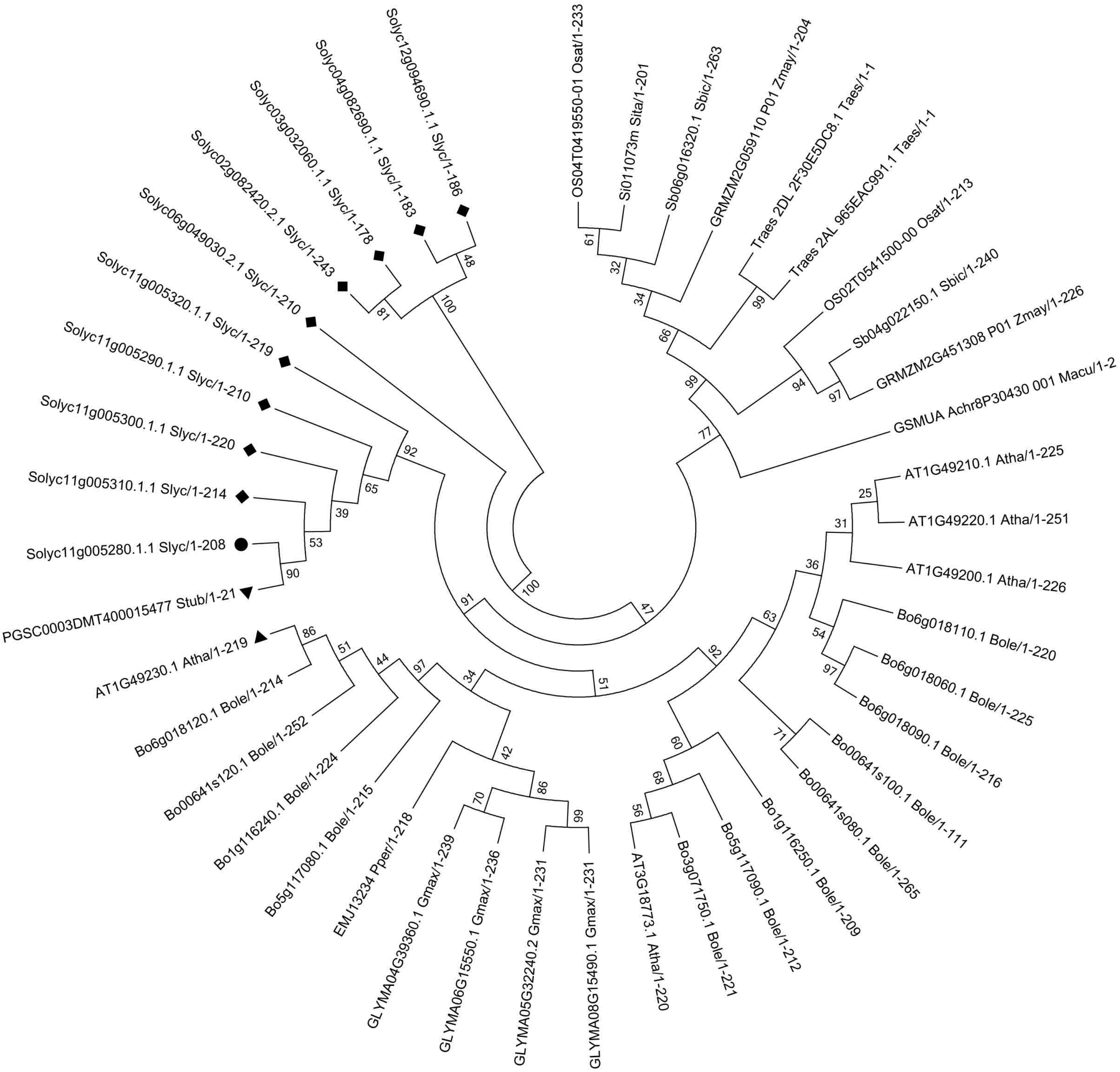

Fig. S1. Evolutionary relationships of ATL78L from different plant species.

GenBank accession numbers of each protein are on the figure.◆, ●, ▼, and ▲ mean a marker of 9 paralogues from Solanum lycopersicum, SlATL78L, the highest homology with ATL78L from Solanum tuberosum and Arabidopsis thaliana, respectively.
