# Supplementary material for: An ATL78-Like RING-H2 Finger Protein Confers Abiotic Stress Tolerance through Interacting with RAV2 and CSN5B in Tomato
Source: Front Plant Sci. 2016 Aug 29;7:1305. doi: 10.3389/fpls.2016.01305 (PMC5002894; doi:10.3389/fpls.2016.01305)
Supplement: Supplementary file 5 [file Image_2.PDF]

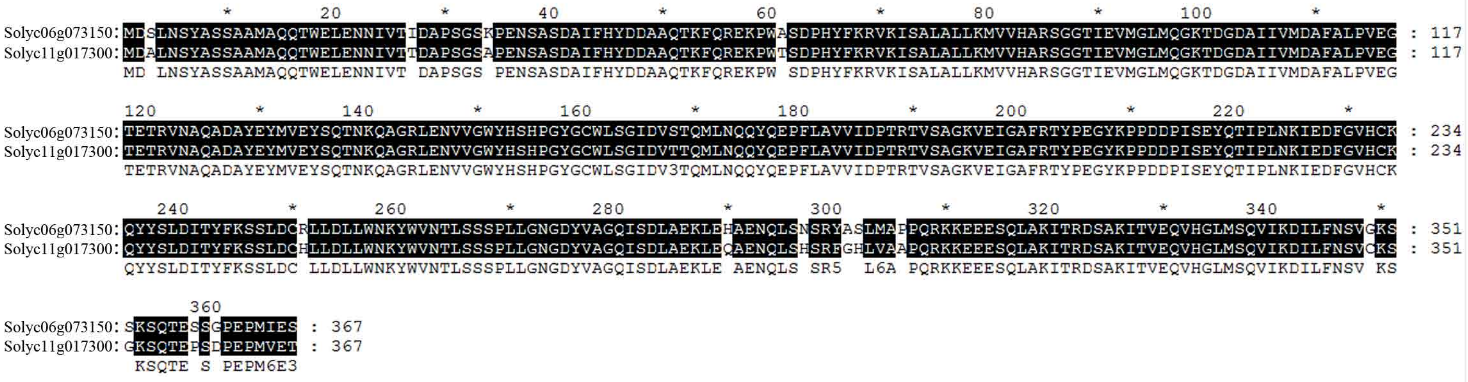

Fig. S2. Alignment of amino acid sequences two CSN5B (Solyc06g073150 and Solyc11g017300) from the database of Sol Genomics Network (https://solgenomics.net/).

The same amino acid sequences are shown on a black background. Sequence alignments were conducted using MUSCLE.
